# Supplementary material for: An online communication skills training program for nursing students: A quasi-experimental study
Source: PLoS One. 2022 May 4;17(5):e0268016. doi: 10.1371/journal.pone.0268016 (PMC9067637; doi:10.1371/journal.pone.0268016)
Supplement: Attachment — Submitted filename: Response to reviewers.docx [file pone.0268016.s003.docx]

**Response to reviewers**

[Reviewer #1]

1. Please submit the list of questions asked as a part of the standard questionnaires used. Please submit literature showing the validity and generalizability of these psychometric/behavioral assessment tools preferably in relation to other tools, if any. This is important to gauge the relative merits of these tools. It is difficult to rely on the study results without solid literature on the validity and generalizability backing the methods section.

**Response**

Thank you for your detailed review. The assessment tools used in the study have been modified and used according to the culture of Korean students (Please refer below). The questionnaire was of an appropriate length and showed a good sense of self-efficacy. We received content effectiveness index (CVI) advice from two experts from the School of Nursing. The CVI score was found to be 0.8.

2. The data tables need to show distribution of each test score as we are dealing with a sample of less than 30 units per control and experiment. Inferences from small samples have a higher risk of getting swayed in one direction or the other.

**Response**

Thank you for your detailed review. The normality of the baseline scores for both groups was tested using the Kolmogorov–Smirnov test, and baseline homogeneity was tested using the Chi-squared test, Fisher’s exact test, and t-test.

3. The study does not give details of when the training was conducted and when the pre-post tests were done. This in the case of an intervention that is online and informative in nature is important as it determines the test results.

**Response**

The pre-tests of the experimental and the control groups were conducted online on October 5, 2020, using Google questionnaire. The NVC program, in which only the experimental group participated, was conducted between October 5 and October 30, 2020. An online post-test using Google questionnaire was conducted for both experimental and control groups on October 30, 2020.

4. The authors also need to clarify how the pre-post surveys were done. Were they online too?

**Response**

Thank you for your review. The pre-post surveys were conducted online using Google forms.

[Reviewer #2]

1. How did you select your sample size?

**Response**

We referred to previous studies related to NVC (Yang, J., & Kim, S. (2020). Effects of a nonviolent communication-based training program for inpatient alcoholics in South Korea. Perspectives in Psychiatric Care).

The sample size to achieve the study objectives was determined using the G*Power 3.1 Program; the minimum sample size was calculated to be 42, with 21 in the experimental group and 21 in the control group (effect size 0.80, significance .05, power .80, two-tailed test). Considering a potential 30% withdrawal rate, 56 students were enrolled. One control group participant dropped out, resulting in 28 participants in the experimental group and 27 in the control group. The effect size was established to be 0.87 in a Korean study that analyzed NVC-based interventions (Han, M., & Lee, K. (2017). Effects of communication ability enhancement program for nursing students in Korea: A systematic review and meta-analysis. The Journal of Korean academic society of nursing education, 23(1), 15–26.).

2. Provide few more lines about research randomizer and G power program to make it more comprehensible for the readers.

**Response**

In this study, participants were assigned to experimental and control groups using the Research randomizer (<https://www.randomizer>.org/) program. The number of groups (two) was entered into the program, and the total number of people (56) for the group was entered. Subsequently, the participants were assigned to the experimental and control groups based on the numbers assigned to them by the program.

3. Who designed NVC-CST program. The authors mentioned the program facilitator but I found lack of any information about who designed the program and what is the foundation of that training program.

**Response**

NVC-CST was developed by the main researcher (Jeongwoon Yang). Also, NVC-CST is a program developed based on NVC (Nonviolent Communication), which was developed by Marshall Rosenberg. It is a specific conversational model that focuses on two components essential to building mature relationships: honest expression and empathic listening (Rosenberg, Marshall B. (1983). A Model for Nonviolent Communication. Philadelphia PA: New Society Publishers.).

4. Authors mentioned some causes (including inefficient communication) of premature resignation of newly graduate nurses, however I am interested to know why authors focused only one reason.

**Response**

I agree with your opinion that, there are various factors such as the working environment, wages, and communication conflicts in the turnover of new nurses. It is impossible to solve structural problems such as the working environment and wages with individual efforts. However, conflict resolution through the acquisition of communication skills is possible through individual efforts. Therefore, this study emphasized communication skill training.
